# A reference database enabling in-depth proteome and PTM analysis of mouse immune cells

**DOI:** 10.1038/s41597-025-04829-9

**Published:** 2025-04-10

**Authors:** Devon Siemes, Hannah Voss, Federica Benvenuti, Francesca Simoncello, Dominik Kopczynski, Bente Siebels, Hartmut Schlüter, Laxmikanth Kollipara, Albert Sickmann, Daniel Robert Engel, Olga Shevchuk

**Affiliations:** 1 Department of Immunodynamics, Institute for Experimental Immunology and Imaging, University Duisburg-Essen, Essen, 45141 Germany; 2 Cellular Immunology Laboratory, International Centre for Genetic Engineering and Biotechnology (ICGEB), Trieste, Italy; 3 Institute for Analytical Chemistry, University of Vienna, Währingerstrasse 38, Vienna, 1090 Austria; 4 Section Mass Spectrometry and Proteomics, Center for Diagnostics, University Medical Center Hamburg-Eppendorf, Hamburg, Germany; 5 Leibniz-Institut für Analytische Wissenschaften-ISAS-e.V., Dortmund, 44139 Germany

**Keywords:** Immunology, Proteomics

## Abstract

Spectral libraries fulfill multiple functions in biological and analytical applications. For biologists, these libraries provide a valuable resource to verify the presence and abundance of proteins or pathways within a selected cell type thus determine the feasibility of further experiments. Despite advances, existing libraries are incomplete and provide researchers only a limited amount of information. To address this, we introduce the reference database - Spectral Library of Immune Cells (SpLICe), a resource covering B-cells, CD4 and CD8 T-cells, macrophages and dendritic cells containing nearly 9,000 protein groups and 110,346 proteotypic peptides. Additionally, the database provides data on > 20,000 post-translationally modified proteotypic peptides (oxidation, phosphorylation, methylation, acetylation, deamidation and N-glycosylation) across the selected immune cell populations. SpLICe supports the quantification of more than half of total murine proteins annotated by UniProtKB/Swiss-Prot, enabling monitoring of selected proteins or pathways from Reactome pathways and Gene Ontology databases. The platform provides relative protein abundances and supports the generation of targeted mass spectrometry assays by identifying and scoring proteotypic peptides.

## Background & Summary

Mass spectrometry (MS)-based proteomics has become an indispensable technology in biological and biomedical research. The application of unbiased ‘discovery’ proteomics, such as ‘shotgun’ proteomics, now enables genome-scale coverage and quantification of proteins, including their post-translational modifications (PTMs) and splice variants. Understanding signaling and metabolic pathways in the cells of interest requires clarity regarding the presence and reliability of detection of specific proteins, which could be improved by peptide reference libraries. To facilitate this, comprehensive spectral libraries have been developed for selected cell lines^1^, human tissues, covering more than 14,000 proteins^2^, and several bacterial human pathogens, including *E.coli*^3^ and Mycobacterium species^4,5^. For primary cells, only one study on a human T-cell spectral library was published so far^6^. Regarding model organisms, a recent development introduced a large murine spectral library encompassing over 11,000 proteins and 240,000 proteotypic peptides. This library was derived from nine murine tissue samples and a murine L929 cell line^7^. Additionally, deep tissue-specific spectral profiling of a murine sepsis model serves as the source of information for biologists^8^.

However, for researchers focusing on proteins and pathways from specific immune cell subsets, the available information is limited. Furthermore, no information is available on immune cell-specific PTM profiles, that are having a relevant impact on the disclosure of cell type, function, and intracellular signaling.

In response to this need, we introduce the Spectral Library of Immune Cells (SpLICe). This comprehensive resource covers five main cell populations and comprises 8,697 protein groups, 110,346 proteotypic peptides and 128,635 spectra. SpLICe supports the quantification of more than half of total murine proteins annotated by UniProtKB/Swiss-Prot. In addition, SpLICe provides information on >20,000 post-translationally modified (PTM) proteotypic peptides, including oxidation, phosphorylation, monomethylation, deamidation, acetylation, and glycosylation.

The platform enables monitoring the presence of selected proteins or pathways of interest through Reactome pathways, GO Biological Process, GO Molecular Function, or GO Cellular Component analysis and provides information about the relative abundance of protein. In addition, the platform supports the generation of targeted mass spectrometry assays by identifying available proteotypic peptides and evaluating their suitability for targeted MS analyses via an integrated scoring system. This resource is valuable for investigations focused on immune cell characterization and quantification within the murine system, making it an essential tool for advancing immunological research.

## Methods

### Primary cell culture

Mouse bone marrow-derived dendritic cells (BMDCs) and BM-derived macrophages (BMDMs) were generated from bone marrow like described before^9^. BMDCs were generated in vitro from BM of mice using GM-CSF. DCs were cultured at a concentration of 1.5 × 10^6^ cells/mL for 7 days using IMDM supplemented with 10% FBS, 50 *μ*M 2-mercaptoethanol (Gibco), 1% gentamicin, complemented with 30% supernatant of GM-CSF produced from J558 cell line. Cells were used for experiments between days 6 and 8. CD11c^+^ DCs were enriched from total spleen cells using the CD11c microbeads, mouse (Miltenyi Biotec). The purity of population was checked by CD11c-PE staining. BMDMs were generated in vitro from BM of C57BL/6 WT mice using M-CSF. BM-derived cells were cultured at a concentration of 1 × 10^6^ cells/mL in non-treated 10 cm Petri dishes (Thermo Fisher Scientific) for 7 days using L929 conditioned medium: RPMI 1640 supplemented with M-CSF (30% L929 cell supernatant), 10% FBS, plus 1% penicillin/streptomycin, 1 mM sodium pyruvate. Cells were used for experiments between days 6 and 8. The purity of total BMDMs population were checked by F4/80-A488 (Clone MCA497, AbDSerotec, 1:25) and CD11b-APC (Clone M1/70, BioLegend 1:200). L929 cells (M-CSF-producing cells) were grown for 1 week in RPMI 1640-supplemented 10% FBS plus 100 *μ*g/mL penicillin, 100 *μ*g/mL streptomycin, 2 mM l-glutamine, 10 mM HEPES, 1 mM sodium pyruvate, and 50 *μ*M *β*-mercaptoethanol. Cell-free supernatant was then harvested from the confluent monolayer, 0.22 *μ*m filtered, and kept at  − 20 °C until use.

### Isolation of murine lymphocytes

To obtain CD4, CD8, and B-cells, spleens from 10-week-old female BC57BL/6 mice were mechanically homogenized through a 40-*μ*m cell strainer (Corning) into single-cell suspensions. Following centrifugation, 1 mL of ACK Lysing Buffer (Gibko, Ref.A10492-01) was added to the pellet, and the mixture was incubated for 5 minutes on ice. If erythrocytes were still visible after the first incubation step, the lysis procedure was repeated, and the incubation time was maintained. Subsequently, cells were stained with biotinylated CD3 antibody (clone 145-2C11; BioLegend, 1:100) for 30 minutes on ice. The cells were washed once with 10 mL of cold PBS containing 2% FBS via centrifugation at 1300 rpm for 5 minutes at 4°C. Following the wash step, the cells were incubated in the dark on ice for 30 minutes with the following antibodies: CD4-BV785 (clone GK1.5, BioLegend, 1:200), CD8a-APC (clone 53-6.7, BioLegend, 1:200), CD19-PE (clone 1D3, BD, 1:200), and Streptavidin BV510 (BioLegend, 1:200) in PBS 2% FBS. The cell sorting was performed on ARIA (Becton Dickinson). Sorted cells were washed in 50 mL of ice-cold PBS for 10 min, 300g at 4 °C, and redissolved in 1 mL of ice-cold PBS for transfer in LoBind tubes. The purity of cells was checked by the same antibodies.

### Sample preparation

10^7^ of BMDCs or BMDMs, 2 × 10^7^ of B-cells and 5 × 10^6^ of CD4- and CD8 T-cells was resuspended in 100-200 *μ*L of lysis buffer, i.e., 50 mM Tris-HCl (pH 7.8) 150 mM NaCl, 1% SDS supplemented with complete mini-EDTA free protease inhibitor (Roche, Penzberg), ultrasonicated 3 times, 1 min each, put on ice to cool down between the cycles. The lysate was centrifuged for 30 min at 20.000g at 4C and the protein concentration in supernatant was determined by BCA assay according to the manufacturer’s instruction. Approximately 100 *μ*g of proteins were reduced in 10 mM DTT for 30 min at 56 °C and further alkylated in 30 mM of Iodoacetamide (IAA) for 30 min at 37 °C in the dark. The remaining IAA was quenched with 30 mM dithiothreitol (DTT) for a further 15 min at 37 °C. Sample cleanup and on-filter proteolysis were performed using filter-aided sample preparation (FASP) as described previously^10^. After 16 h at 37 °C, the tryptic peptides were eluted with 200 *μ*l of 25 mM NH4HCO3 (pH 7.8), acidified to a final pH < 3.0 with 10% trifluoroacetic (TFA) and transferred to HLB 96-well *μ*Elution Plate, 30 *μ*m (Oasis, Water) for desalting according to manufacturer’s instructions. The eluted peptides were dried under vacuum (SpeedVac), re-solubilized in 0.1% TFA, and 1% of the digest was quality controlled^11^.

### Generation of spectral libraries

For generation of spectral library, 20 *μ*g of peptides were fractionated on a 0.3 × 150 mm 3.5 *μ*m Zorbax 300 extend-C18 (Agilent) with a flow rate of 10 *μ*L/min and a gradient length of 95 min ranging from 3–45% of B (84% ACN in 10 mM ammonium formiate, pH 8). Twelve fractions were collected over the gradient, acidified with 10% TFA, vacuum-dried and reconstituted with 15.5 *μ*l of 0.1% TFA. All spectral libraries fractions were analyzed using an Ultimate 3000 nano-RPLC system coupled to QExactive HF (both Thermo Scientific). Peptides were pre-concentrated on a 100 *μ*m × 2 cm C18 trapping column for 5 min using 0.1% TFA with a flow rate of 20 *μ*L/min followed by separation on a 75 *μ*m × 50 cm C18 main column (both Acclaim Pepmap nanoviper, Thermo Scientific) with 180 min LC gradient ranging from 3–35% of B (84% ACN in 0.1% FA) at a flow rate of 250 nL/min. The mass spectrometer was operated in data-dependent acquisition (DDA) mode. Precursor scanning was performed, covering an m/z range of 300 to 1500 m/z on an Orbitrap mass analyzer at a resolution of 60.000 using the polysiloxane ion at m/z 371.10124 as lock mass. Ions were accumulated for 120ms or to an ion count (AGC Target) of 3 × 10^6^. For each MS1 scan, the top 15 most abundant precursors (TopN = 15), with a charge state between 2 and 4, were isolated and fragmented at a normalized collision energy (nce) of 27. MS2 scans were performed on an Orbitrap mass analyzer at an Orbitrap resolution of 15.000 covering a scan range from 200 to 2000 m/z. At MS2 level, ions were injected for 200ms or up to a AGC Target of 5 × 10^4^ ions. Selected precursor ions were isolated using quadrupole with a 1.2 m/z window considering a dynamic exclusion of 20s.

### Data analysis

MS raw data was processed with Proteome Discoverer software 3.1.0.638 (Thermo Scientific) and searched against a target/decoy mouse UniProt database (downloaded on 29th of October 2024, containing 17221 target sequences), using the integrated Sequest HT algorithm. The search parameters were: Precursor and fragment ion tolerances of 10 ppm and 0.02 Da for MS and MS/MS, respectively; trypsin as enzyme with a maximum of 2 missed cleavages; carbamidomethylation of Cys as fixed modification; oxidation of Met was selected as variable modification. Peptides were excepted with a length of between 6 and 150 amino acid. PSM validation was performed trough Percolator, using a q-value — based concatenated Target/Decoy selection. For identification, an FDR < 0.01 was required at the peptide and protein levels. Only Peptides identified via PSM matching were considered. For additional information on immune cell-specific biologically relevant post-transnational modification (PTM) profiles, separate database searches were performed using, deamidation (N, Q), monomethylation (K, R), phosphorylation (S, T, Y), acetylation (R) and acetylation of the N-Terminus, as additional variable modifications. For the identification of peptides with associated N-Asparagine-linked Glycans, pGlyco (Version 3, pFind, Beijing, China) was used^12^. Glycopeptides were searched against same UniProt database (downloaded on 29th of October 2024, containing 17221 target sequences) and the curated pGlyco N-Mouse database (downloaded on November 1, 2024). Peptides were accepted at a mass of 600 to 4000 Da and a length of 6 to 40 amino acids. A maximum of two modifications per peptide were allowed. Fixed and variable modifications at N-Glycans were disabled. For identification, FDR < 0.01 was required at the N-Glycan and peptide levels. Cumulative count plots, histogram, rank plot, and clustermap were created using matplotlib (3.9.3)^13^ and seaborn (0.13.2)^14^. Hierarchical clustering was calculated using protein-wise min-max-scaled values and the complete linkage algorithm from scipy (1.14.1)^15^ on the pairwise Euclidean distance matrix. The upset plot was generated using the python package UpSetPlot (0.9.0)^16^.

### SpLICe interface

The SpLICe interface was created in Python using datavzrd (v.2.45.1)^17^, which links data-tables and utilizes vega-lite (v.5) to deploy interactive and interconnected visualizations in a lightweight html file. The ranking of proteotypic peptides for targeted assays was generated using information extracted from PeptidePicker, Picky, and Skyline recommendations^18–20^. Several parameters were considered in the ranking process: presence of the peptide in the spectral library (+1), uniqueness within the mouse proteome (+2), length of 6-25 amino acids (+1), and lack of missed tryptic cleavages (+1). Peptides without Methionine or Cysteine were given +1, as were those lacking Tryptophan or Glutamic acid at the N-terminus. Peptides with no Proline-Proline, Aspartic acid-Proline, or Aspartic acid-Glycine sequences were also awarded +1 for each. Additionally, peptides without sequences containing more than three consecutive Serine residues were given +1. PTMs were only included in the interface, if the respective peptide was identified as proteotypic.

## Data Records

The mass spectrometry proteomics data have been deposited to the ProteomeXchange Consortium via the PRIDE partner repository and can be accessed via https://identifiers.org/pride.project:PXD059683^21,22^. The SpLICe repository, i.e. raw tables, python code, and supplementary tables, has been uploaded to Zenodo and can be accessed via 10.5281/zenodo.14629899^23^.

## Technical Validation

### Preparation of immune cell subsets

**Fig. 1:**
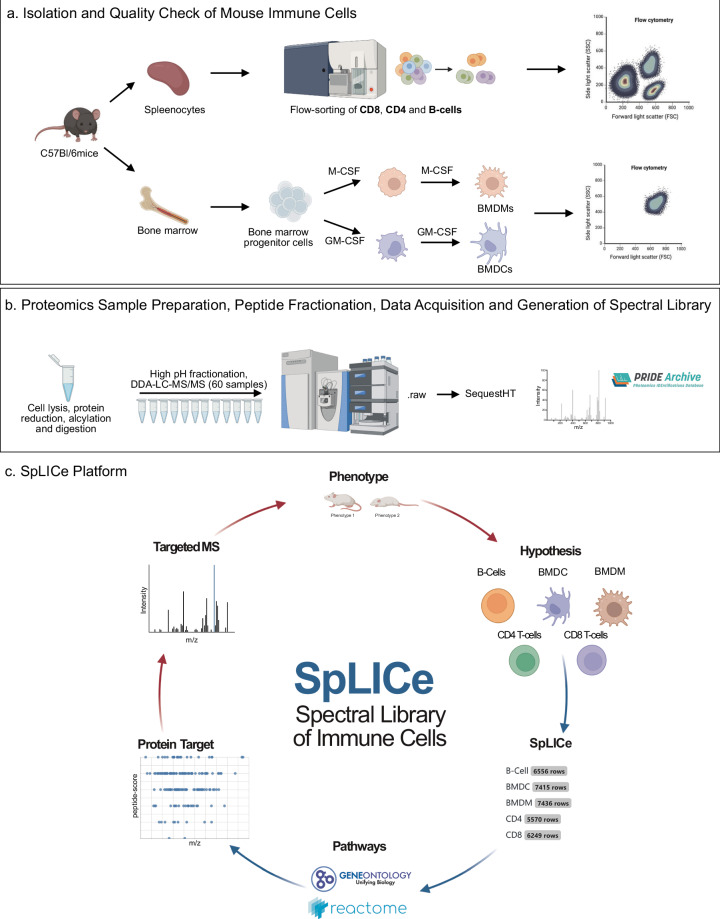
The workflow to generate the primary immunological spectral libraries. (**a**) Key procedures encompassed the isolation of mouse splenocytes followed by purification of CD8 T-cells, CD4 T-cells, and B cells, along with the isolation of bone marrow and subsequent differentiation into BMDMs and BMDCs; (**b**) Proteomic sample preparation, peptide fractionation, and LC-MS/MS data acquisition; (**c**) Individual spectral library generation for distinct immune cells integrated in data analysis platform facilitating efficient extraction of information about pathways, associated proteins and corresponding peptides from a HTML file. Partially created with BioRender.com.

Isolation and sample preparation of immune cell subsets of high purity is an essential step for generating specific spectral libraries and estimating relative or reference values for proteins in proteomics targeted assays (DIA, PRM and MRM methods). To achieve this, we applied flow cytometry cell sorting to obtain purified CD8 T-cells, CD4 T-cells, and B-cells from mouse spleen (Fig. 1a). The purity of the populations was at least 98.5% for spleen lymphocytes. Bone marrow-derived macrophages (BMDMs) and bone marrow-derived dendritic cells (BMDCs) were selected as they are the most common in vitro model systems and prepared from bone marrow differentiation by M-CSF and GM-CSF, respectively. The purity of obtained BMDMs was 99.8%, and for BMDCs it was 81% (Table 1). We analyzed each proteome in its steady state by pooling four mice for splenocytes cell sorting (Fig. 1a). To generate a comprehensive immunological spectral library, proteins from each cell type were trypticaly digested and subjected to high pH fractionation. Twelve fractions per cell type were analyzed by DDA in LC-MS/MS gradients and searched using Sequest HT searching engines (Fig. 1b). At a peptide and protein false discovery rate (FDR) of 1%, we identified almost 9,000 proteins with an average of 6,556 proteins per cell type. Based on our calculation, we obtained approximately 100 pg of peptides from a single BMDM or BMDC, 20 pg of peptides per cell for CD4 and CD8 T-cells, and 10 pg per single B-cell (Table 1).

**Table 1:** Characteristics of immune spectral libraries.

Cell type	Source	Purity	Protein amount/cells	Protein, N	Peptide, N	Spectra, N
CD4 T-cells	Spleen	98.6%	20 *μ*g/10^6^	5570	34001	140164
CD8 T-cells	Spleen	99.5%	20 *μ*g/10^6^	6249	56882	231474
B-cells	Spleen	98%	10 *μ*g/10^6^	6556	45950	210084
BMDMs	BM	99.8%	100 *μ*g/10^6^	7436	84440	312872
BMDCs	BM	81%	100 *μ*g/10^6^	7415	71229	262422

### Characteristics of the SpLICe

**Fig. 2:**
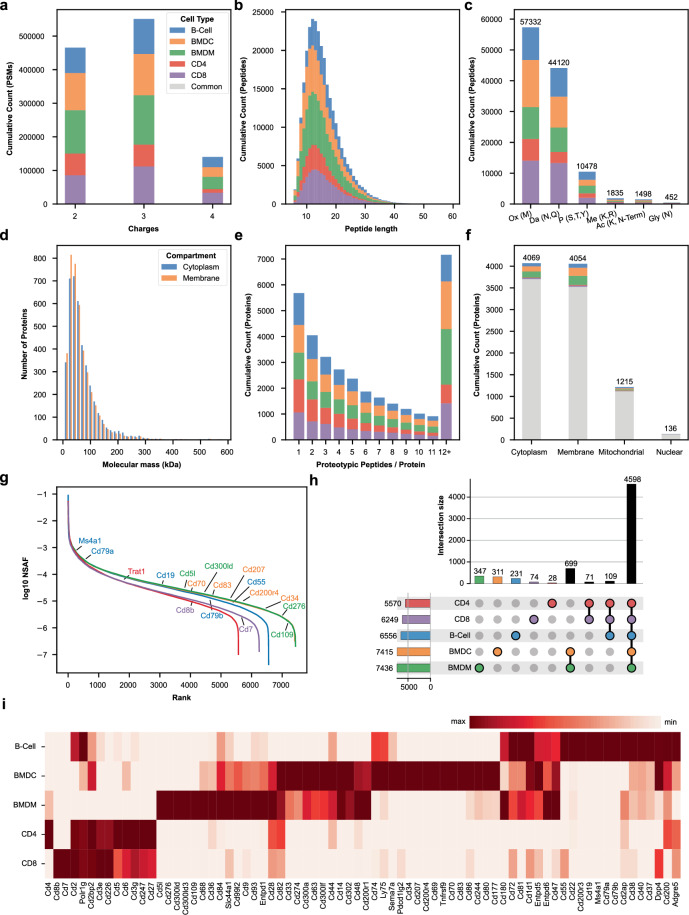
Coverage and characteristics of the Spectral Libraries of Immune Cells (SpLICe). (**a**) Stacked histogram of precursor charges for all cell type libraries. (**b**) Peptide length distribution and (**c**) number of peptides with oxidation (Ox), deamidation (Da), phosphorylation (P), methylation (Me), acetylation (Ac), or glycosilation (Gly) PTMs. (**d**) Molecular mass distribution of identified proteins, (**e**) the distribution of proteotypic peptides per protein, and (**f**) their subcellular distribution. (**g**) Relative abundance of each protein as indicated by the normalized spectral abundance factor (NSAF) across respective cell types. Characteristic cluster differentiation (CD) markers are specified for each proteome. (**h**) UpsetPlot displaying the number of proteins unique to each cell type and all possible combinations of intersections. The core proteome shared among all cell types comprises N=4598 proteins. (**i**) Clustermap indicating selected cluster of differentiation (CD) markers.

Spectral libraries serve multiple functions in biological and analytical applications. For biologists, these libraries provide a valuable resource to verify the presence and abundance of specific proteins or pathways within a selected cell type, thereby determining the feasibility of conducting further experiments. In mass spectrometry, spectral libraries significantly enhance the sensitivity and accuracy of peptide identification in tandem mass spectrometry (MS/MS) analyses^24^. Moreover, these libraries facilitate the selection of peptides, proteins and pathways for targeted assays such as Parallel Reaction Monitoring (PRM) or Multiple Reaction Monitoring (MRM). The restricted availability of high-quality libraries constitutes its drawbacks. To ensure the high-quality of acquired data we explored the following main characteristics: the measured peptides in our libraries featured between 2+ to 4+ charge per precursor (Fig. 2a). After processing the PSMs by removing redundant identifications, the resulting peptide groups show complementary distributions for the peptide length and number of experimentally induced and natural occurring modifications per peptide for all cell types (Fig. 2b,c). Peptide lengths range between 6 and 59, and the sum of modified peptides from all cell lines ranges from 452 glycosylated peptides to 57332 oxidized peptides. The proteins were identified using the peptide groups with 99.9% being between 5.0 kDa and 584 kDa (Fig. 2d). Each protein has a median of 5 proteotypic peptides with 83% having more than 1 (Fig. 2e). The measured proteins come from a variety of compartments featuring 4069 cytoplasmic, 4054 membrane-associated, 1215 mitochondrial and 136 nuclear proteins (Fig. 2f). In addition to protein abundance profiles, the diversity of post-transnational modifications (PTMs) across cell types is crucial for cell-specific function. Disclosing them enables scientists to understand cellular processes and disease-related alterations. PTM information in cell-type spectral libraries can be used to enhance the identification of modified peptides in immune cell-related proteomics experiments. Furthermore, databases of immune cell type-specific PTM profiles can help biologists to verify the cellular identity of samples. SpLICe integrates immune cell-specific profiles of >20,000 modified proteotypic peptides, including oxidation (M) (N = 23,893 peptides, covering 5,892 proteins), phosphorylation (S, T, Y) (N = 5,237 peptides, covering 2,290 proteins), monomethylation (K, R) (N = 1,060 peptides, covering 792 proteins), deamidation (N, Q) (N = 25,342 peptides, covering 6,294 proteins), acetylation (K) (N = 885 peptides, covering 738 proteins), and glycosylation (N) (N = 330 peptides with 278 plausible N-glycan structures, covering 167 N-glycan compositions across 180 proteins). (Fig. 2c). To ensure high reliability of PTM data, only modifications at proteotypic peptides, identified with an FDR < 0.01 were integrated into the SpLICe. For N-glycoproteomic data, were complex N-glycan structure is deconvoluted via additional glycan spectrum matching at the MS2 level a additional FDR < 0.01 was for the identification of N-glycan compositions was required.

### Immunological markers of the SpLICe

The estimated relative abundance of proteins in the immune cells was calculated using the normalized spectral abundance factor (NSAF). NSAF determines the proportional contribution of individual proteins to the total proteome^25^. Based on the NSAF values, the abundance of proteins from all cell populations span six orders of magnitude. Similarly to previously published studies in ultra-deep proteomics, a relatively small number of proteins constitute a high proportion of the total protein mass^26,27^. The minimal number of proteins (5570) was found in CD4 T-cells, and the maximum (7436) was detected in BMDMs. Most of the proteins (4598 proteins, 53%) were commonly expressed in all analyzed cell types, whereas 0.3% (28 proteins for CD4 T-cells and up to 4% (347 proteins for BMDMs) were expressed in a cell type-specific manner (Fig. 2h). Selection and clustering of identified CD-markers show clear grouping of known and new surface proteins (Fig. 2i). Additionally, cells of the same origin, e.g., T-lymphocytes, display similar expression of Cd27, Cd247, Cd226, Cd5, Cd6, Cd3, and Cd2, but could be differentiated by Cd4 and Cd200, presented mainly on CD4-T cells. Cd8b and Cd7 are present exclusively on CD8 T-cells. To provide a comprehensive overview of possible surface markers, we analyzed our deep-coverage spectral libraries for the exclusively expressed proteins of the immune cells (Fig. 2h). In addition to well-characterized markers, we found a set of CD molecules expressed only in one cell type (Table 2).

**Table 2:** Cluster differentiation (CD) markers unique for analyzed immune cell type.

Cell type	Protein accession	CD marker	Description
CD4 T-cells	P56484 Q8VCN6	CCr8 Cd99	C-C chemokine receptor type 8 Cd99 antigen
CD8 T-cells	Q61476 P50283 P10300	Cd55b Cd7 Cd8b	Complement decay-accelerating factor transmembrane isoform T-cell antigen Cd7 T-cell surface glycoprotein Cd8 beta chain
B-cells	P11911 P15530 P25918 P19437 Q61475	Cd79a Cd79b Cd19 Cd20 Cd55	B-cell antigen receptor complex-associated protein alpha chain B-cell antigen receptor complex-associated protein beta chain B-lymphocyte antigen Cd19 B-lymphocyte antigen Cd20 Complement decay-accelerating factor, GPI-anchored
BMDMs	Q8R422 Q8VE98 Q8VCH2 Q8BTP3 Q9QWK4	Cd109 Cd276 Cd300ld Cd200r5 Cd5l	Cd109 antigen Cd276 antigen CMRF35-like molecule 5 Cell surface glycoprotein Cd200 receptor 5 Cd5 antigen-like
BMDCs	Q5UKY4 Q64314 Q6XJV4 Q8VBX4 O88324 O55237	Cd200r3 Cd34 Cd200r4 Cd207 Cd83 Cd70	Cell surface glycoprotein Cd200 receptor 3 Hematopoietic progenitor cell antigen Cd34 Cell surface glycoprotein Cd200 receptor 4 C-type lectin domain family 4 member K Cd83 antigen Cd70 antigen

## Usage Notes

### Spectral Library

To facilitate the design of proteomic experiments targeting immune cells, we have developed SpLICe, an interactive spectral library embedded in an easily accessible HTML file (Fig. 1c). By linking data tables to interactive visualizations using datavzrd, SpLICe allows users to:

1. obtain information about the relative abundance of proteins within cells
2. collect comprehensive peptide information for each protein
3. explore the presence of signaling and metabolic pathways within cells of interest
4. investigate immune cell specific PTM patterns
5. determine specific protein expressions and markers for cluster differentiation (CD) within the cells of interest

For each immune cell type, the table contains the following information: UniProt protein accession and description, gene symbol, number of unique peptides per protein, and relative abundance of proteins. Additionally, the table includes columns for Reactome and Gene Ontology pathways from MSigDB (v2024.1) mouse-specific collection, allowing for a phenotype-driven inquiry. Cell type specific protein and peptide data are connected to a plot highlighting the peptides with corresponding ranking. To ensure the selection of high-quality, reliable peptides for targeted mass spectrometry assays we ranked peptides using information extracted from PeptidePicker, Picky, and Skyline recommendations^18–20^. The peptide information is in a detail view that can be accessed through the + sign next to the Accession-ID, featuring the sequence, m/z-value, charge and peptide scores for each peptide. In addition, the protein sequence with PTM sites annotated is situated in the detail view if any PTMs for that protein were found for this immune cell. The table for each of the cell type can be accessed from a dropdown menu on the top left. Clicking on the UniProt accession ID of any entry will open the Uniprot website for further information on that protein. Filtered tables may be retrieved for in-depth downstream analysis. We plan to extend SpLICe by incorporating additional significant leukocyte population spectral libraries under both steady-state and activated conditions. Spectral libraries related to immune cells from other users could also be integrated.

### Reproducibility

Using the code and the supplementary data supplied with SpLICe, all figures, numbers and SpLICe itself can be reproduced or modified. After setting up the Python environment with “environment.yml”, the manuscript data can be generated by executing the file “manuscript.py” and the tables required to build SpLICe can be prepared with “splice.py”. The SpLICe interface can be generated with datavzrd and the command “datavzrd –overwrite-output rendered.yml -o SpLICe”.

## Data Availability

The Python code used to post-process the protein, peptide and spectral data and to generate SpLICe is published on Zenodo and accessible with the DOI 10.5281/zenodo.14629899. Along with the code, an environment.yml file is provided, listing the packages used and their respective versions. In addition, the base protein, peptide and spectral tables extracted from Proteome Discoverer and the required additional tables are included.
